# Effective strategies for childhood obesity prevention via school based, family involved interventions: a critical review for the development of the Feel4Diabetes-study school based component

**DOI:** 10.1186/s12902-020-0526-5

**Published:** 2020-05-06

**Authors:** Christina-Paulina Lambrinou, Odysseas Androutsos, Eva Karaglani, Greet Cardon, Nele Huys, Katja Wikström, Jemina Kivelä, Winne Ko, Ernest Karuranga, Kaloyan Tsochev, Violeta Iotova, Roumyana Dimova, Pilar De Miguel-Etayo, Esther M. González-Gil, Hajnalka Tamás, Zoltán JANCSÓ, Stavros Liatis, Konstantinos Makrilakis, Yannis Manios, on behalf of the Feel4Diabetes-study group, Yannis Manios, Greet Cardon, Jaana Lindström, Peter Schwarz, Konstantinos Makrilakis, Lieven Annemans, Ignacio Garamendi, Yannis Manios, Kalliopi Karatzi, Odysseas Androutsos, George Moschonis, Spyridon Kanellakis, Christina Mavrogianni, Konstantina Tsoutsoulopoulou, Christina Katsarou, Eva Karaglani, Irini Qira, Efstathios Skoufas, Konstantina Maragkopoulou, Antigone Tsiafitsa, Irini Sotiropoulou, Michalis Tsolakos, Effie Argyri, Mary Nikolaou, Eleni-Anna Vampouli, Christina Filippou, Kyriaki Apergi, Amalia Filippou, Gatsiou Katerina, Efstratios Dimitriadis, Jaana Lindström, Tiina Laatikainen, Katja Wikström, Petteri Hovi, Jemina Kivelä, Päivi Valve, Esko Levälahti, Eeva Virtanen, Greet Cardon, Vicky Van Stappen, Nele Huys, Lieven Annemans, Ruben Willems, Samyah Shadid, Peter Schwarz, Patrick Timpel, Konstantinos Makrilakis, Stavros Liatis, George Dafoulas, Christina-Paulina Lambrinou, Angeliki Giannopoulou, Lala Rabemananjara, Maria Stella de Sabata, Winne Ko, Ignacio Garamendi, Luis Moreno, Fernando Civeira, Gloria Bueno, Pilar De Miguel-Etayo, Esther Mª. Gonzalez-Gil, María L. Miguel-Berges, Natalia Giménez-Legarre, Paloma Flores-Barrantes, Aleli M. Ayala-Marín, Miguel Seral-Cortés, Lucia Baila-Rueda, Ana Cenarro, Estíbaliz Jarauta, Rocío Mateo-Gallego, Violeta Iotova, Tsvetalina Tankova, Natalia Usheva, Kaloyan Tsochev, Nevena Chakarova, Sonya Galcheva, Rumyana Dimova, Yana Bocheva, Zhaneta Radkova, Vanya Marinova, Yuliya Bazdarska, Tanya Stefanova, Imre Rurik, Timea Ungvari, Zoltán Jancsó, Anna Nánási, László Kolozsvári, Csilla Semánova, Éva Bíró, Emese Antal, Sándorné Radó, Remberto Martinez, Marcos Tong

**Affiliations:** 1 Department of Nutrition and Dietetics, School of Health Science and Education, Harokopio University, 70 El Venizelou Ave, 176 71 Kallithea, Athens, Greece; 2 Department of Nutrition and Dietetics, School of Physical Education, Sport Science and Dietetics, University of Thessaly, Trikala, Greece; 3 Department of Movement and Sports Sciences, Faculty of Medicine and Health Sciences, Ghent University, Ghent, Belgium; 4 Department of Public Health Solutions, National Institute for Health and Welfare, Helsinki, Finland; 5 International Diabetes Federation European Region, Brussels, Belgium; 6 Department of Pediatrics, Medical University Varna, Varna, Bulgaria; 7 Department of Diabetology, Clinical Center of Endocrinology, Medical University Sofia, Sofia, Bulgaria; 8 Growth, Exercise, NUtrition and Development (GENUD) Research Group. Instituto Agroalimentario de Aragón (IA2), Instituto de Investigación Sanitaria Aragón (IIS Aragón), University of Zaragoza, Zaragoza, Spain; 9 Centro de Investigación Biomédica en Red de Fisiopatología de la Obesidad y Nutrición (CIBERObn), University of Zaragoza, Zaragoza, Spain; 10 Institute of Nutrition and Food Technology. Department of Biochemistry and Molecular Biology II, Center of Biomedical Research, University of Granada, Granada, Spain; 11 University of Debrecen, Department of Family and Occupational Medicine, Debrecen, Hungary; 12 National and Kapodistrian University of Athens, Athens, Greece

**Keywords:** Obesity prevention, Type 2 diabetes prevention, Primary school children, Families, School based intervention

## Abstract

**Background:**

Although there are many interventions targeting childhood obesity prevention, only few have demonstrated positive results. The current review aimed to gather and evaluate available school-based intervention studies with family involvement targeting dietary, physical activity and sedentary behaviors among primary schoolchildren and their families, in order to identify the most effective strategies.

**Methods:**

Studies published between 2000 and January 2015 were retrieved from scientific electronic databases and grey literature. The databases used included MEDLINE/PubMed, Web-of-Science, CINAHL and Scopus. Included studies had to be experimental controlled studies and had duration over 1 school year, had family involvement, combined PA and dietary behaviors and were implemented in school setting. A complementary search was executed to update the review to cover the period from February 2015 to January 2019.

**Results:**

From the studies examined (*n* = 425), 27 intervention programs (33 publications) fulfilled the inclusion criteria. Among these, 15 presented significant effect on weight status and/ or overweight/ obesity or clinical indices, 3 presented significant effect on most energy balance-related behaviors (EBRBs) while 9 presented significant effect on some/few EBRBs or determinants. Strategies implemented in effective interventions were: teachers acting as role-models and being actively involved in the delivery of the intervention, school policies supporting the availability of healthy food and beverage choices and limiting unhealthy snacks, changes in the schoolyard, in the recess rules and in the physical education classes to increase physical activity, and involving parents in the intervention via assignments, meetings, informative material and encouraging them to improve the home environment. Use of incentives for children, social marketing techniques, collaboration with local stakeholders were found to increase effectiveness. Programs that focused only on educational sessions and material for parents, without promoting relevant environmental and policy changes, were found to be less effective. Cultural adaptations have been suggested to increase the intervention’s acceptance in specific or vulnerable population groups.

**Conclusions:**

Several effective strategies were identified in the reviewed programs. Outcomes of the current review were taken into account in developing the Feel4Diabetes-intervention and summed up as recommendations in the current work in order to facilitate other researchers designing similar childhood obesity prevention initiatives.

## Background

Childhood obesity is a growing world-wide health problem. It is estimated that about 170 million children are currently with overweight globally [1]. In US children, obesity prevalence has increased from 6% in 1980 to 17% in 2008 [2] and to 18.5% in 2016 [3]. In Europe, the trend of childhood obesity is following the same pattern, with its prevalence increasing across most European countries [4], especially in low-to-middle-income countries (LMICs) and in vulnerable groups [5, 6].

Being overweight or obese has serious health consequences, especially for children. Having a high body mass index (BMI) is a major risk factor for diseases such as cardiovascular disease, type 2 diabetes and cancer later in life [7, 8]. These diseases, also known as non-communicable diseases (NCDs), can cause premature mortality as well as long-term morbidity. Due to the significant increase in the prevalence of obesity and the serious public health consequences, obesity is considered one of the most important public health challenges of the twenty-first century [9, 10].

Early childhood is considered to provide a unique opportunity to establish lifestyle behaviors such as healthy eating habits, physical activity and limited sedentary time that will promote health and minimize the risk of obesity. This is further supported by the fact that, as a results of these behaviours, childhood obesity tracks into adulthood with overweight preschool children being more likely to become overweight adults in comparison to their normal-weight peers [11–15]. Thus, interventions promoting such healthy behaviors in childhood provide a key strategy in the prevention of obesity, since it has been shown that treating obesity in adulthood poses more difficulties than changing lifestyle habits earlier on [16].

To date, many programs have been developed in order to prevent obesity in children. The vast majority of these programs use schools for the implementation of interventions [17]. Schools are considered an important setting for intervening in children’s obesity related behavior, for a number of reasons: (i) primary school education is compulsory for all children in most countries and reaches all children with different backgrounds (ii) children spend a significant part of their daily life at school, usually consuming one or two meals per day; (iii) schools offer physical education classes and provide opportunities for physical activity during recess; (iv) schools offer a structured environment where interventions can be easily applied/ fit, (v) implementers can reach many children in a relatively short time via schools; (vi) teaching staff can significantly facilitate and contribute to the delivery of the intervention, thus increasing the intervention’s sustainability [18]. Despite the aforementioned advantages, the overall impact of school-based interventions is questionable and generalizable recommendations cannot be easily extracted [2].

For the needs of the European multicenter Feel4Diabetes-study the current study examined interventions that have been implemented in the school setting and focused on the promotion of healthy eating and physical activity and the reduction/interruption of sedentary behavior aiming to prevent childhood obesity. The Feel4Diabetes-study was implemented in the overall population in low/middle-income countries (Bulgaria, Hungary), in low socio-economic areas in high-income countries (Belgium, Finland) and in countries under austerity measures (Greece, Spain). More information on this project can be found in the literature [19]. The aim of the current review was to identify the most effective strategies in improving health behaviors and tackling obesity in primary school-aged children with special emphasis given to low socioeconomic status and vulnerable groups that Feel4Diabete-study specifically targeted, taking into account their increased risk for obesity [9, 20] and type 2 diabetes [21]. The Feel4Diabetes project was developed using the PRECEDE-PROCEED model as the theoretical basis. The current review served as a part of the PRECEDE phase of this specific theoretical model and its outcomes were utilized in the development of the Feel4Diabetes school- and community-based intervention.

## Methods

### Search strategy

The bibliographic search strategy focused on articles published in peer-reviewed, English language journals, published from 2000 to January 2015, when the Feel4Diabetes-intervention was developed. A complementary search was executed to update the results for the publication to cover the period from February 2015 to January 2019 following the same methodology. The databases used included MEDLINE/PubMed, Web-of-Science, CINAHL and Scopus. Citations in reviews were also used. The PICO key terms used for conducting the literature search are presented in Supplementary Table 1.

### Selection criteria

To be included, studies had to be original experimental controlled studies with school-based interventions aiming to prevent obesity in primary school-aged children from any country, published between 2000 and January 2019, in English. Additional inclusion criteria were applied based on recent literature [18, 22]. These criteria were, namely: a) duration of > 1 school year (i.e. at least 6 months), b) family involvement, either by contacting parents via their children with the use of newsletters, etc. or by having meetings with them, c) combination of physical activity and dietary behaviors, d) implementation of the school-based intervention by school personnel (i.e. healthcare professionals working at schools or teachers). These criteria were applied in order to avoid repetition of already proven to be effective strategies and take the existing literature a step further.

### Exclusion criteria

Exclusion criteria included interventions implemented in preschools, early childcare programs, adolescents or after-school programs, descriptive or analytic studies, reviews on surgical or pharmaceutical treatments, literature reviews, opinions or editorials, reports published as meeting abstracts only, reviews of causal relationships between obesity and related factors, articles reporting study design and/or process evaluation only as well as papers focused on physiological, molecular or genetic research and papers focused on morbidities (such as kidney disease and diabetes) in which obesity is a comorbidity.

### Selection strategy and data extraction procedures

**Fig. 1:**
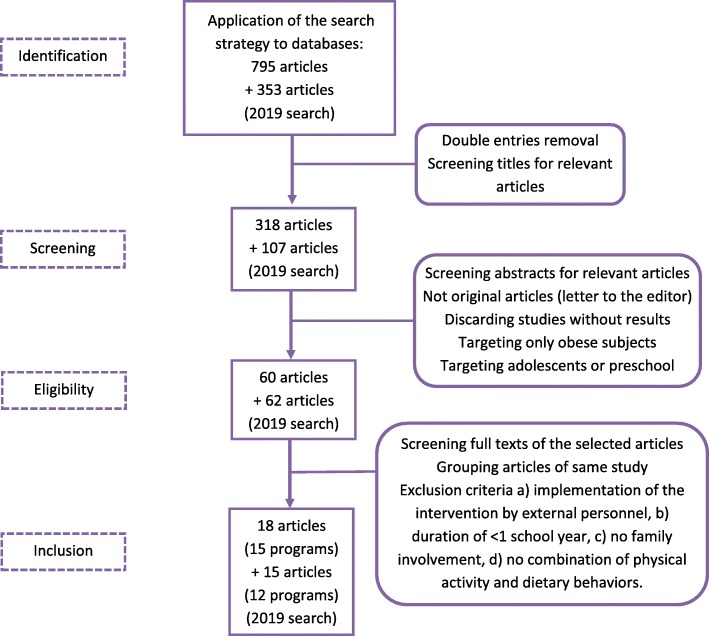
Flowchart of the publication identification process. Effective strategies in childhood obesity prevention interventions at the primary-school setting: A narrative review for the development of the Feel4Diabetes-study intervention

The flowchart of the publication identification process is presented in Fig. 1. Data from the included studies were extracted using a standardized form. Extracted data included: author, year of publication, project name, country, participant data, intervention duration, theory used and strategies implemented as described in the methodology of the relevant publications, outcomes as well as follow-up period if available. In addition, the setting in which the intervention was implemented was also extracted in order to identify strategies that proved to be effective in low-SES areas or in vulnerable groups. A summary table of the selected studies was constructed and the clinical significance of the results was evaluated for each study, to facilitate interpretation of the effectiveness of intervention. The scoring is marked in the summary table as follows:

- (+++) significant effect on weight status and/ or overweight/ obesity or clinical indices.
- (++) significant improvement in most important target behaviors.
- (+) significant improvement in some/few secondary target behaviors.
- (−) no effect.

## Results

The studies included in the current analysis have been summarized in Table 1 outlining the target population, intervention strategies and design, measures, outcomes, and findings relevant to obesity prevention. The interventions in table have been arranged alphabetically based on first author’s name but with clustering the publications of same projects together.

**Table 1:** Overview of the publications included in the review

Publication	Program name	Country	Population group	Setting	Intervention duration	Intervention Approach & Theoretical framework	Post-trial follow-up duration	Outcomes & Effectiveness score
Angelopoulos et al., 2009 [19]	CHILDREN study	Greece	13 schools 646 pupils 5th grade (Mean ± SD: 10.3 ± 0.4 years)	–	1 school year	School and home environment (availability of healthy choices) Policies (Children’s and teachers in-class-material, Enjoyable fitness classes, Schoolyards open after school hours) Parents engagement (Meetings to support children’s energy-balance related behaviors (EBRBs), Increasing parental support, Overcoming the barriers in accessing physical activity (PA) areas)) Teachers acting as role-models ***Theory of Planned Behavior***	–	Significant favorable effect on fruits consumption and fats/oils and sweets/beverages consumption. Significant favorable effect on BMI (*P* = 0.047) could be explained by the changes in fruit and fats/oils intake. (+++)
Benjamins et al., 2010 [20]	–	Chicago	2 schools 581 students 1st-8th grade	Jewish schools	2 school years	Formation of a wellness council Writing of a wellness policyHealth education (via health lessons) Physical education (by providing funding to increase PA, gender separated swimming lessons) School environment (removing vending machines, healthy lunches, skim-milk promotion, fruit of the week program) Family involvement (school events, meetings, newsletters) Staff wellness (role-models, private dietary consultations, educational sessions) ***The Centers for Disease Control (CDC) ecological model-based Coordinated School Health Program***	–	Significant favorable effect n the percentage of older students regularly meeting physical activity guidelines. Few changes in attitudes, other behaviors, or environmental factors were seen. (++)
Bhave et al., 2017 [21] ^a^	SYM-KEM Study	India	865 children 3rd and 4th grades (age range: 7.7–9.6 years).	Academically competitive Indian school	2 school years	Increased extra- and intra-curricular physical activity sessions Daily yoga-based breathing exercises Making physical activity a ‘scoring’ subject Nutrition education Healthier school meals Removal of fast-food hawkers from the school environs Health and nutrition education for teachers, pupils and families ***Theory Framework not mentioned***	5 years	After five years the intervention had significant favorable effect on running, long-jump, sit-up and push-up tests. Significant favorable effect on sedentary time (watching TV and studying), active play time and fruit consumption. Significant favorable effect on waist circumference. No effect on BMI or the prevalence of overweight/ obesity. (+++)
Brandstetter et al., 2012 [22]	URMEL-ICE	Germany	945 children 2nd grade (Mean ± SD: 7.6 ± 0.4 years)	–	1 school year	29 teaching modules, 2 short exercise blocks per day 6 family homework lessons Parental material Teachers training ***Social Cognitive theory***	–	No statistically significant effect of the intervention on BMI. Significant favorable effect on waist circumference and subscapular skinfold thickness before additional adjustment for individual time lag between baseline and follow-up. The intervention group revealed a higher percentage of children with an improvement and a lower percentage with a worsening of the health-relevant behaviors compared to the control group. (+++)
Cohen et al., 2014 [23]	The CHANGE study	California, Kentucky, Mississippi and South Carolina, USA	8 schools432 children grades 1-6th (Mean ± SD: 8.6 ± 1.5 years)	rural low-SES areas	2 school years	Food service component (healthier choices) Educational curriculum every week Parent and community outreach components throughout the school district to promote the healthy lifestyle changes encouraged during and after the school day. ***Theory Framework not mentioned***	–	Significant favorable effect on vegetables consumption and on combined fruits and vegetables consumption Favorable trend toward more fruit consumption (not significant) No significant effects on whole grains, legumes, dairy, potatoes/potato products, saturated fat, added sugars, or dietary fiber consumption. (+)
De Coen et al., 2012 [24]	POP project	Belgium	31 schools, 1102 children 3–6 years (Mean ± SD: 5.0 ± 1.3 years)	–	2 school years	Family involvement via educational strategies, newsletters, tips, recipes Development of an active playground Implementation of health-related physical education Environmental and policy changes to increase availability of healthy options Community stakeholders, local policy and media (meetings, brochures) involvement ***Socio-ecological model***	–	Significant favorable effect on BMI Z-score in the low-socioeconomic status (SES) intervention community. (+++) only in low-socioeconomic status (SES) groups.
Foster et al., 2007 [25]	–	Philadelphia, USA	10 schools 1349 children 4th - 6th grade (Mean ± SD: 11.1 ± 1.0 years)	–	2 school years	School Nutrition Policy Initiative: School self-assessment (school rating and action plan for change) Staff training (10 h) Nutrition education (50 h) Nutrition policy (cafeteria offering only healthy options, removal of vending machines) Social marketing (Slogans, character/ hero, raffle tickets) Parent outreach (meetings, workshops) ***Social marketing and other theories***	–	The intervention resulted in a 50% reduction in the incidence of overweight. Significantly fewer children in the intervention schools than in the control schools became overweight after 2 years. The prevalence of overweight was lower in the intervention schools. No differences were observed in the incidence or prevalence of obesity or in the remission of overweight or obesity at 2 years. Significant favorable effect on inactive hours per week and on hours spent on TV on weekdays. (+++)
Jensen et al., 2015 [26]	Copenhagen School Child Intervention Study (CoSCIS)	Copenhagen, Denmark	18 schools 307 children with full data Mean age 6.8 years at baseline	–	3 school years	Two additional physical education (PE) lessons per week Additional education of PE teachers Improvement of schoolyard environment (recess) Parent involvement via newsletters Establishment of school canteens Health education in the curriculum ***Theory Framework not mentioned***		Significant favorable effect on dietary fibre intake Favorable trend on fat and saturated fatty acids intake. Significant favorable effect on the intake of saturated fatty acids among children of mothers with higher education. (+)
Kain et al., 2008 [27]	–	Chile	4 schools 2430 students 1st-8th grade (Mean ± SD: 10.0 ± 2.3 years)	–	2 school years	Teachers’ training Parents’ education via meetings Children’s educational and PE classes Active recess ***Theory Framework not mentioned***	–	Significant favorable effect on obesity prevalence and BMI-Z in both boys and girls (+++)
Kesztyues et al., 2017 [28]^a^	The Baden-Wurttemberg Study “Join the Healthy Boat” program	Germany	1733 children (Mean ± SD: 7.1 ± 0.6 years)	–	1 school year	Teachers’ training All materials were integrated into the regular curriculum Materials for children (e.g. activity breaks) and for parents (e.g. family homework and information material) Translation of the material in order to also reach parents with migration background ***Intervention mapping, social cognitive theory & socio-ecological model***	–	Significant favorable effect on abdominal obesity (+++)
Kobel et al., 2017 [29]^a^				Migration background Sub-sample				Significant favorable effect on fruits & vegetables intake Favorable trend on PA and soft drink consumption (+)
King et al. 2015 [30]^a^	–	rural elementary schools in the southern United States	4 rural elementary School 999 children kindergarten to third grade (Mean age: 7.3 years)	rural, low-socioeconomic status elementary schools	1 school year	Nutrition and health Education SPARK PE curriculum Classroom PA Strengthening school wellness policies Health promotion for teachers and families Promoting family involvement and community partnerships Culminating goal for each school to achieve ‘bronze’ or higher status of the Healthier US School Challenge ***The CDC’s ecological model-based Coordinated School Health Program***		Significant favorable effect on nutrition and physical activity behaviors Significant favorable effect on the percentage of children meeting the nutrition recommendation Significant favorable effect on the percentage of children meeting the physical activity recommendation (++)
Kipping et al., 2014 [31]	AFLY5	South west England	60 schools2221 students 4th grade (Age range: 8-9 years)	–	1 school year	Teacher training Provision of 16 lessons & child-parent interactive homework plans Materials for lessons and homework, and written materials for school newsletters and parents. ***Social Cognitive Theory***	–	No effect on the three primary outcomes (PA, sedentary time, diet) Significant favorable effect on three out of nine of the secondary outcomes, i.e. self-reported time spent in screen viewing at the weekend, self-reported servings of snacks per day, and servings of high energy drinks per day. (+)
Lawlor et al., 2016 [32]^a^				–			–	Significant favorable effect on 3/10 potential mediators, i.e. fruit and vegetable self-efficacy; child-reported maternal limitation of sedentary behavior; and knowledge. (+)
Anderson et al., 2016 [33]^a^				–			1 year post intervention	No effect on mean child-reported screen viewing at the weekend, servings of snacks per day, servings of high-energy drinks per day, servings of high-fat foods per day. (−)
Llargues et al., 2011 [15]	AVall study	Granollers Spain	16 schools500 students 1st grade (Age range: 5-6 years)	–	2 school years	Educational sessions to promote healthy eating habits and physical activity Information session with the parents Distribution of healthy recipes ***Research, Vision, Action and Change (IVAC) methodology***	–	Significant favorable effect on BMI and the prevalence of overweight children Significant favorable effect on the proportion of children that ate a second piece of fruit Significant favorable effect on the consumption of fish. (+++)
Lloyd et al., 2017 [34]^a^	Healthy Lifestyles Programme (HeLP)	South West of England	32 schools 1324 children (Age range: 9–10 years)	–	1 school year	Dynamic & interactive activities e.g. Physical activity workshops Education sessions delivered by teachers with short homework tasks Drama sessions Goal setting to modify behavior with parental support and one-to-one discussions with the project’s coordinators Extensive stakeholder involvement ***Intervention mapping***	–	No significant effects on anthropometric or physical activity outcomes Significant favorable effect on the adjusted means of the Food Intake Questionnaire scores (both weekly and weekday) for energy-dense snacks and negative food markers (+)
Manios et al., 2002 [35]	–	Crete, Greece	40 schools 5681students 1st grade (Age range: 6-7 years)	–	6 school years	Multicomponent workbooks for students Teaching aids included posters, audio-taped fairy tales for classroom use, workbooks, and teaching manuals Non-competitive activities Meetings with parents Booklet distribution ***Social Cognitive theory***		Significant favorable effect on biceps skinfold Significant favorable effect on total energy intake, consumption of total fat and saturated fat Significant favorable effect on time devoted to leisure time physical activity (+++)
Manios et al., 2006 [36]							4 years post intervention	Significant favorable effect on MVPA levels for males and on males meeting the recommendations for physical activity (+)
Manios et al., 2006 [37]								Significant favorable effect on total cholesterol, LDL-cholesterol, HDL-cholesterol and total cholesterol: HDL-cholestrol ratio Significant favorable effect on leisure-time physical activities and BMI No effects on fitness and dietary indices examined. (+++)
Mårild et al., 2015 [38]^a^	IDEFICS study	Belgium, Cyprus, Estonia, Germany, Hungary, Italy, Spain, Sweden	7406 children (age 2–9.9 years) of the 16,228 participating	–	1 school year	Sustainable change in health behaviors and in the community environment in cooperation with political leaders, teachers and stakeholders. ***Intervention Mapping***	–	No effects on insulin, HOMA-IR, CRP or the MetS score Significant favorable effect on fasting glucose, a pattern driven by three of the eight countries and more pronounced in children of parents with low education. Significant unfavorable effect on HbA1c and waist circumference increased more and blood pressure less in the intervention regions. (+) only in glucose levels but otherwise contradicting results.
Mihrshahi et al., 2017 [39]^a^	Good Start Program	Maori and Pacific Islander communities living in Queensland	375 children (6–19 years)	Maori and Pacific Islander communities living in Queensland	1 school year	Class activities focused on one message each term related to healthy eating and physical activity using methods such as cooking sessions and cultural dance. ***Theory Framework not mentioned***	–	Quantitative uncontrolled pre-post design. Significant favorable time effect on knowledge of correct servings of fruit and vegetables, knowledge of sugar and caffeine content of common sugar-sweetened drinks, recognition of the consequences of marketing and upsizing, and the importance of controlling portion size, knowledge of physical activity recommendations, as well as the importance of physical activity for preventing heart disease and improving self-esteem Significant favorable time effect on some attitudes to vegetables and sugar-sweetened drinks and the reported intake of vegetables (+)
Pablos et al., 2017 [40]	Healthy Habits Program (HHP)	Valencian Community, Spain	2 schools 158 children (10–12 years)	–	1 school year	Free extracurricular activity: brief 10-min talk about healthy habits Physical exercise session targeting fun, inclusion and cooperation, and safety (themed games) All the sessions were led by the same specifically-trained teacher. A worksheet designed by the research team was given to be completed at home (29 in total) Three 45-min talks for parents and teachers about healthy habits for school children. ***Theory Framework not mentioned***		Significant favorable effect on triglycerides, blood glucose and VO2max, breakfast habits and quality of diet, the prevalence of normal levels for total cholesterol, blood pressure and BMI. (+++)
Plachta-Danielzik et al., 2007 [41]	KOPS study	Kiel, Germany	32 schools1764 students (Age: 6 years)	–	1 school year	Teachers training on a structured nutrition education program. Health messages were given to children, parents, and teachers, conveyed as nutrition fairy tales, interactive games, and by preparing a healthy breakfast. Six nutrition units performed during 2 to 3 weeks within the second term of the first school year. After each unit, running games were offered for 20 min on the schoolyard.Parents were informed during a parental school meeting. ***Theory Framework not mentioned***	4 years post intervention	No effect on mean BMI Significant favorable effect on the prevalence of overweight and obesity in children from families with high socioeconomic status and marginally significant in children of normal-weight mothers. (+++) only in high-SES groups.
Plachta-Danielzik et al., 2011 [42]							8 years post intervention	No effect on mean BMI, lifestyle and blood pressure Significant favorable effect on the 8-year change in BMI- standard deviation scores (SDS) in high SES groups (+++) only in high-SES groups.
Rush et al., 2014 [43]^a^	Project Energize	New Zealand	193 primary schools 4804 children (Age range: 6-11 years)	42% Māori, the indigenous people of New Zealand		Encourage healthy behaviors daily Healthy choices availability and decrease the availability of high energy/ low nutrient foods Increase the awareness of healthy choices Consistent nutrition messages in all aspects of school and community interaction e.g., healthy fundraising options Encourage lunchtime physical activity at least twice a week Raise awareness of incidental activity opportunities at home and school ***Theory Framework not mentioned***		Significant favorable effect on the combined prevalence of obesity and overweight and BMI, physical fitness in both boys and girls, both indigenous Maori and non-Maori children, and across SES. (+++)
Sacchetti et al., 2015 [44]^a^	SAMBA project	Bologna, Italy	11 classes 234 school children (Age range: 8-11 years)	–	3 school years	Training modules for teachers Training modules for instructors of local sport societies Educational activities in class Free and structured games inside the school and in the open. Cookery workshops and sensory courses for parents and teachers. Moments of movement with parents in free time: homework Dog walking & Home-school routes on foot/by bike Creation of didactic materials (recipes, leaflets, DVDs, food pyramids) ***Precede-Proceed Model***		Significant favorable effect on weight, the percentage of children who consumed an adequate mid-morning snack, the percentage of children who consumed snacks and drinks after the dinner and the percentage of those who consumed five or more portions of fruits and vegetables daily. (+++)
Sahota et al., 2001 [45]	APPLES	Leeds, UK	10 schools 634 students (Age range: 7-11 years)	–	1 school year	Teacher training Modification of school meals Development of school action plans (Individualized on school level based on their needs) Parental involvement ***Theory Framework not mentioned***	–	Significant favorable effect on vegetable consumption No effect on physical activity or sedentary behavior (+)
Shofan et al., 2011 [46]	–	Israel	2 schools 108 students 4th–6th grade (Age range: 9-11 years)	–	2 school years	8 nutritional education lessonsDouble physical education hours (intense aerobic activity designed to increase the aerobic component by 50%)Regular parents meetings, once a month for one hour per session for 10 months a year, Encouragement of healthy dietary habits. ***Theory Framework not mentioned***	–	Significant favorable effect on the average BMI percentile Significant favorable effect on weight for boys No effect on weight or BMI in girls. (+++)
Spiegel et al., 2006 [47]^a^	WAY program	USA	69 classes 1013 students 4th&5th grade (Age range: 9-11 years)	–	1 school year	Intervention teachers participated in workshops on the intervention and received program materials.Family involvement through activities and discussionsClass modules ***Theory of Reasoned Action***	–	Significant favorable effect on BMI, the consumption of fruits and vegetables and physical activity levels (+++)
Springer et al., 2012 [48]	Marathon Kids	Texas, USA	511 students4th–5th grade Mean age: ~ 10 years	Low-SES area	1 school year	Miles trackingNumber of F&V tracking Kick-off and Final Mile Run in public venues with celebrities-mayors-professional athletes as hosts/ public role-modelsCommunity events/ festivalsT-shirts-medals at the end, logo, stickers, advertising on buses & scheduled time for walking/ running at school ***Ecological Model by Sallis*** **et al*, 2006***	–	Significant favorable effect on the mean time of running in past 7 days the mean fruit and vegetable consumption, athletic identity self-concept, PA outcome expectations, and PA and fruit and vegetable consumption self-efficacy (++)
Weber et al., 2017 [49]	SMS. Sei schlau. Mach mit. Sei fit. [‘Be smart. Join in. Be fit.’]	Germany	Four 3rd and 4th grade classes (70 children) as intervention & 6 classes (125 children) as control group Mean age: ~ 9 years	migration background	1 school year	2 additional exercise lessons weekly (“Fitness für Kids”) and 10 nutrition lessons per school year. In the trial, parental involvement was limited to participation in evening meetings and accompanying their children to extra-curricular activities. ***Theory Framework not mentioned***	–	Significant favorable effect on fitness and motor skill driven by higher improvements in 5 of the 8 test items, i.e., obstacle race (speed), standing long jump (strength), sit-ups (strength), stand and reach (mobility), and 6 min run (endurance), independently of confounders. No effects on dietary knowledge and consumption frequencies. (+)
Xu et al., 2015 [50]^a^	CLICK-Obesity Study	Nanjing City, Mainland China	8 urban primary schools grade 4 1125 students Mean age: ~ 10 years		1 school year	Classroom curriculum, School environment support, Family involvement and fun programs/events) together with routine health education ***Theory of Triadic Influence (TTI) and the CDC’s ecological model-based Coordinated School Health Program***		Marginal (non-significant) favorable effect on mean BMI value. Significant favorable effect on likelihood to decrease their BMI by 0.5 kg/m2 or above, increase the frequency of jogging/ running, decrease the frequency of TV/computer use and of red meat consumption, change commuting mode to/from school from sedentary to active mode, and be aware of the harm of selected obesity risk factors. (++)

^a^The studies were included at the 2nd literature search (February 2015–January 2019), after the Feel4Diabetes intervention development

Abbreviations: *PE* physical education; *PA* physical activity; *EBRBs* energy-balance related behaviors; *CDC* Centers for Disease Control; *SES* socioeconomic status; *SDS* standard deviation scores; *PE* physical education

### General description of findings

Using the aforementioned search strategy 1148 publications were identified. After removing duplicates and a first title screening 425 articles remained. These 425 articles were screened based on their abstract, in order to discard studies without yet published results, those targeting specific populations (e.g. only obese children or patients), preschoolers or adolescents. Of the 122 articles that remained, full-texts were extracted and reviewed based on the inclusion/ exclusion criteria. Finally, 27 intervention programs (33 publications) fulfilled the criteria and were included in the current review.

Most of the included interventions (*n* = 19) were implemented in high income countries (HICs), however a few of them (*n* = 8) were specifically focused on vulnerable population groups or low-to-middle income countries (LMICs), while two of the studies implemented in HICs took vulnerable groups under consideration using extra analysis [23]. The sample size varied across the studies, ranging from 108 to 7406 participants.

### Outcome and effectiveness of school-based interventions on weight status and energy balance related behaviors

Out of the 27 intervention studies (33 publications) included in the current review, 15 (55% of included studies) presented significant effect on weight status and/ or overweight/ obesity or clinical indices, 3 (11% of included studies) presented significant effect on most target behaviors while 9 (33% of included studies) presented significant effect on some/few target behaviors. Only a few interventions (*n* = 4) followed-up the participants post-intervention, showing promising long-term effectiveness especially in high-socioeconomic status (SES) groups.

### Intervention strategies

Key strategies have been identified from the effective intervention programs. In studies including various intervention strategies and several outcomes, no direct cause-effect link could be identified, however the strategies or the combination of strategies used in successful interventions were found. Regarding teachers’ involvement, having the teachers trained by health professionals to deliver the intervention, coordinate school-based activities and promote healthy energy-balance related behaviors (EBRBs) during school hours instead of having researchers or other personnel implementing the intervention has been suggested as an effective strategy. In-class material (workbooks, posters, manuals) was used to facilitate the process. Moreover, teachers acting as role-models and getting actively involved in all targeted EBRBs has been highlighted as another effective strategy.

Regarding school policies, several strategies such as the increased water accessibility, the free provision of fruits, the availability of only healthy options in the school cafeteria and the removal of vending machines seem to be efficacious strategies. The formation of a wellness council (by school staff) and a written wellness policy were reported as some more drivers of the intervention’s effectiveness.

The involvement of the family was a prerequisite for the inclusion of studies in the current review. Parents were approached via several methods, i.e. family “homework” assignments, educational/ informative material (newsletters) including healthy recipes and school meetings/ events. In all cases the aim was to encourage changes at the home environment as well in order to further promote the targeted behaviors (e.g. availability of fruit and vegetables) and ensure the continuity of the intervention after school hours.

Several other strategies were also implemented in the childhood obesity prevention interventions that were found to be effective. More specifically, physical activity promotion during recess and the development of an active playground as well as non-competitive, enjoyable activities, promoting whole class participation during physical education (PE) classes and the provision of additional hours to physical activity were shown to be effective strategies. Motivational incentives (e.g. stickers, t-shirts) as well as social marketing techniques targeting children, e.g. slogans, characters, raffle tickets have also been shown to contribute to effectiveness of the interventions. The collaboration with community stakeholders, local community and school policy and media in every aspect of a health promotion project targeting physical activity, healthier diet or both has been also highlighted as an important contributor to the implementation fidelity, and thus effectiveness, of the intervention, also providing the basis for a sustainable approach.

Regarding intervention programs delivered in vulnerable groups and/ or low-SES areas, effective strategies focused on school policy and environmental changes, parental engagement, incentives and interactive activities such as cooking lessons and cultural dance classes, with less emphasis given on the educational part of the intervention. Furthermore, an intervention program particularly targeting Jewish population offered gender separated physical activity classes for pupils, adapting the intervention to the needs of the specific population group thus increasing adherence and effectiveness of the intervention.

### Theoretical frameworks

Various behavioral theories, models and frameworks have been utilized for the development of the studied school-based interventions. More specifically: the Social Cognitive Theory, the Theory of Planned Behavior, the Theory of Reasoned Action, the Ecological model, the Social-Ecological model, the Coordinated School Health Program model developed by the Centers for Disease Control and Prevention (CDC), Social Marketing, Intervention Mapping, the Precede-Proceed Model, as well as the Research, Vision, Action and Change methodology or a combination of the above were used as the theoretical basis for interventions’ development. On the other hand, about half of the projects (11 out of 27) did not mention whether their intervention was based on a theoretical model. Based on the current review, the most effective theories used in the development of health promotion programs in the school setting are the Social Cognitive Theory and the CDC’s ecological model-based Coordinated School Health Program which is commonly used in the US.

## Discussion

The current review aimed to gather and evaluate available school-based, family-involved obesity prevention interventions targeting dietary, physical activity and sedentary behaviors among primary schoolchildren and their families. Moreover, it aimed to identify the most effective strategies in improving those EBRBs and tackling obesity in primary school-aged children with special emphasis given on low-SES and vulnerable groups.

Previous reviews on the same field were used as a basis for the current review and the inclusion criteria applied [18, 22, 24]. In order to avoid repetition of already proven to be effective strategies and in order to extract new meaningful results from the existing literature, the current review goes one step further in the identification of effective strategies by incorporating those already known to be effective as inclusion criteria. From the total number of studies selected 27 intervention studies (33 publications) fulfilled the inclusion criteria and were included in the current analysis.

As previously described, primary schools seem to be the ideal setting for childhood obesity prevention interventions since it offers many opportunities for physical activity promotion and nutrition education and reduction of sedentary behavior through practice, policy, and a supportive environment [18]. This approach requires the active involvement and participation of teachers. Since schools mainly focus on academic achievement, many teachers find it difficult to include extracurricular modules into the already tight schedule. Furthermore, not all teachers are willing to act as champions for a health promotion intervention, especially if their own lifestyle habits do not follow/ agree with the healthy lifestyle messages delivered via the intervention [25]. Strong engagement by the school headmaster/ leadership is crucial in order to achieve engagement of families and teachers [26] and ensure the delivery of the intervention with the highest possible fidelity and thus, effectiveness as well as teachers’ behavior as role-models.

Parental influence on children‘s nutrition and physical activity habits is a well-known determinant of childhood obesity [27]. Based on research indicating that the involvement of parents in addressing nutrition, physical activity and sedentary behavior in children is essential, the current review only included interventions that involved parents [18, 22]. Several effective strategies such as including parents via family “homework” assignments, educational/ informative material (newsletters) and school meetings/ events have been used to involve parents in the intervention. Meetings with parents at school seemed to lead to increased effectiveness in more cases in comparison to written material, i.e. newsletters.

Given that vulnerable groups and/ or people living in low-SES areas are more likely to be overweight or obese, have poorer health outcomes, and tend to benefit less from interventions in comparison with their counterparts from more affluent backgrounds, both developed and developing countries should also target low-income, high-risk groups [28]. Tailoring interventions to fit the needs of a specific population group, as done in the study by Benjamins et al. where gender separated physical activity classes were offered for Jewish pupils, has been shown to be imperative for the intervention’s acceptability and effectiveness [28]. A thorough situational analysis and formative research should be included in the time planning for the development of interventions, especially when targeting schools in low-SES areas and vulnerable groups [28]. The results of the current review are in agreement with previous work highlighting that intervention programs delivered in vulnerable groups and/ or low-SES areas should focus on school policy and environmental changes and interactive activities with less emphasis on the educational part of the intervention [28, 29].

Using a solid theoretical framework as a basis in an intervention’s design is vital to its chances of success, as evidence suggests that such interventions are more effective compared to those that are not based on a theory [30]. Various theoretical frameworks have been utilized by the included interventions, while almost half of the projects did not mention the use of a theoretical model, a fact that might have limited their effectiveness. CDC’s ecological model-based Coordinated School Health Program which is commonly used in the US was one of the two more popular theories used in the development of health promotion programs in the current review. Its significant effect might derive from the fact that among several targets and in contrast to most frameworks it also aims at improving employee wellness. This characteristic might have made a difference for its popularity and effectiveness. One the other hand, the Social Cognitive Theory is a commonly used basis for interventions design. In the current review, even though it was popular in the current review, its effectiveness varied. Last but not least, integrating policies and/ or environmental changes, such as increased availability and accessibility of healthy choices and removal of unhealthy choices but also the collaboration with community stakeholders, have been shown to be a high impact intervention strategies [31, 32], especially when targeting lower SES groups.

Several outcomes have been considered to assess the interventions’ effectiveness in various studies. Both behaviors and clinical outcomes where considered in the included studies, however the interventions did not always succeed in having an effect on clinical outcomes. The most commonly used and assessed behavioral outcome was the consumption of fruits and vegetables and many interventions succeeded in increasing their consumption followed by physical activity, which proved to be more difficult for the interventions to significantly improve. On the other hand, BMI was the most commonly used clinical outcome assessed. Several interventions succeeded in improving BMI, while some that did not showed an effect on other important adiposity measures, i.e. waist circumference of skinfold thickness. Still, having a significant effect on clinical outcomes is not always feasible even in well-designed interventions, especially in this age group.

## Strengths and limitations

The results of the current work should be interpreted in consideration of the study’s strengths and limitations. In the current review studies in languages other than English were omitted, which could limit the generalization of the results in other countries, especially low-to-middle income countries (LMICs). Studies without published outcomes were excluded as it was not possible to evaluate their efficacy. Although the benefits of single health behavior change interventions vs. multiple health behavior change interventions remain unclear [33], the present review only included interventions targeting dietary, physical activity and sedentary behaviors simultaneously. On the other hand, the current review goes beyond the existing literature by applying specific inclusion criteria that have been linked to increased effectiveness and thus, focusing on the most effective programs to extract the best intervention strategies, contributing to the updating of the literature.

## Conclusions

School-based interventions are vital in the prevention of the globally rising childhood obesity. Many interventions have shown promising results, which were supported by a number of effective and high-impact strategies. Multiple strategies are used in effective interventions, highlighting the fact that a one-size-fits-all approach is not applicable in childhood obesity prevention intervention programs development and that many different strategies can be effective. However, future school-based obesity prevention interventions should build on already successful intervention strategies while also addressing and integrating culture specific strategies. Including long-term follow-up measurements to assess the efficacy of school-based interventions will facilitate the identification of the most effective strategies in the long-term.

Further studies are needed to elucidate the effectiveness of specific strategies aiming at long duration interventions. Both the development and the implementation methodology of the research, as well as the barriers, challenges and possible facilitators should be encouraged to be thoroughly recorded and published in order to inform the scientific community on the feasibility and sustainability of implementing interventions in real life situations.

### Recommendations for future school-based obesity prevention interventions

Apart from the already mentioned recommendations that have been used as inclusion criteria in the present review, several strategies have been proven efficacious, i.e.:

- The use of a suitable theoretical framework should be considered in the intervention design
- Interventions should be adapted to the needs of the targeted population group(s)
- Collaboration with community stakeholders, local policy and media should be targeted in order to increase intervention’s acceptability and sustainability
- School teachers should act as role-models and get actively involved in all targeted behaviors
- The teaching staff and school personnel should promote the availability and accessibility of healthy snacks and water and monitor the provision only of healthy food options by the school cafeteria/canteen and attend to the removal of unhealthy choices and vending machines, if any
- Schools should be encouraged to form a wellness council (school staff and/or parents’ association) and write a wellness policy
- Families should be targeted via educational/ informative material (newsletters), family “homework” assignments or school meetings/ events for the children and their families in order to change the home environment to promote the targeted behaviors (e.g. availability of fruit and vegetable, as well as other healthy food items/snacks)
- School yards should be accessible after school hours and physical activity during recess should be promoted
- PE-instructor/school teachers should develop and promote non-competitive, enjoyable activities, promoting whole class participation
- Social marketing techniques, interactive activities and motivational incentives can be utilized for children.
- In low-SES areas or in migrant population groups, interventions should focus on school policy and environmental changes, parental engagement and interactive team activities, rather than the educational part of the intervention.

## Supplementary information

**Additional file 1: Table 1** PICO keywords.

## Data Availability

Not applicable.

## Abbreviations

BMI: Body mass index
CDC: Centers for Disease Control and Prevention
EBRBs: Energy balance related behaviors
HICs: High Income Countries
LMICs: Low-to-Middle Income Countries
SES: Socio-economic status
PE: Physical education
