# Supplementary material for: Effective strategies for childhood obesity prevention via school based, family involved interventions: a critical review for the development of the Feel4Diabetes-study school based component
Source: BMC Endocr Disord. 2020 May 6;20(Suppl 2):52. doi: 10.1186/s12902-020-0526-5 (PMC7201517; doi:10.1186/s12902-020-0526-5)
Supplement: Supplementary file 1 — Additional file 1: Table 1 PICO keywords. [file 12902_2020_526_MOESM1_ESM.docx]

**Supplementary Table 1.** PICO keywords

| **Population 1** | **Population 2** | **Intervention** | **Comparison** | **Outcome eating** | **Outcome PA** | **Outcome SB** |
| --- | --- | --- | --- | --- | --- | --- |
| children OR childhood OR school OR schoolchildren OR “school children” OR schoolaged OR “school aged” | parent OR caregiver | “Health promotion program” OR “Health promotion programme” OR “health promotion intervention” OR “school setting” OR “school based intervention” OR “school based program” OR “school based programme” OR “school program” OR “school programme” OR “school intervention” |  | food habits OR “dietary habits” OR eating habits OR “food intake” OR “food choices” OR feeding OR dietary OR dieting OR fruits OR vegetable OR “Fruit drinks” OR fruit juice OR fruit juices OR “sugar sweetened” beverage OR soft drink OR soft drinks OR fizzy drink OR “fizzy drinks” OR whole grains OR confectionary OR snacking OR snacking habits OR breakfast OR fast food OR meal frequency OR meal size OR portion size OR unhealthy diet | “Total PA” OR MVPA OR VPA OR total physical activity OR moderate to vigorous physical activity OR vigorous physical activity OR physical activity OR “physical inactivity” OR sports OR sports participation OR “active transport” OR active commuting OR leisure activity OR walking OR “aerobic exercise” OR “outdoor play” OR exercise | sedentary behavior OR sitting behavior OR sitting time OR “domestic activities” OR total sedentary time OR computer use OR video games OR tablet use OR smartphone use OR computer time OR gaming OR “screen time” OR reading OR TV viewing OR TV child room OR television viewing OR video viewing OR eating watching TV |
